# Developmental Variability in Autism Across 17 000 Autistic Individuals and 4000 Siblings Without an Autism Diagnosis: Comparisons by Cohort, Intellectual Disability, Genetic Etiology, and Age at Diagnosis

**DOI:** 10.1001/jamapediatrics.2022.2423

**Published:** 2022-07-18

**Authors:** Susan S. Kuo, Celia van der Merwe, Jack M. Fu, Caitlin E. Carey, Michael E. Talkowski, Somer L. Bishop, Elise B. Robinson

**Affiliations:** 1 Stanley Center for Psychiatric Genetics, Broad Institute of MIT and Harvard, Cambridge, Massachusetts; 2 Center for Genomic Medicine, Massachusetts General Hospital, Harvard Medical School, Boston; 3 Analytic and Translational Genetics Unit, Massachusetts General Hospital, Harvard Medical School, Boston; 4 Department of Neurology, Massachusetts General Hospital, Harvard Medical School, Boston; 5 Department of Psychiatry, University of California, San Francisco; 6 Department of Epidemiology, Harvard T. H. Chan School of Public Health, Boston, Massachusetts; 7 Department of Psychiatry, Massachusetts General Hospital, Harvard Medical School, Boston

## Abstract

**Importance:**

Presence of developmental delays in autism is well established, yet few studies have characterized variability in developmental milestone attainment in this population.

**Objective:**

To characterize variability in the age at which autistic individuals attain key developmental milestones based on co-occurring intellectual disability (ID), presence of a rare disruptive genetic variant associated with neurodevelopmental disorders (NDD), age at autism diagnosis, and research cohort membership.

**Design:**

The study team harmonized data from 4 cross-sectional autism cohorts: the Autism Genetics Research Exchange (n = 3284; 1997-2015), The Autism Simplex Collection (n = 694; 2008-2011), the Simons Simplex Collection (n = 2753; 2008-2011), and the Simons Foundation Powering Autism Research for Knowledge (n = 10 367; 2016-present). The last sample further included 4145 siblings without an autism diagnosis or ID.

**Participants:**

Convenience sample of 21 243 autistic individuals or their siblings without an autism diagnosis aged 4 to 17 years.

**Main Outcomes and Measures:**

Parents reported ages at which participants attained key milestones including smiling, sitting upright, crawling, walking, spoon-feeding self, speaking words, speaking phrases, and acquiring bladder and bowel control. A total of 5295 autistic individuals, and their biological parents, were genetically characterized to identify de novo variants in NDD-associated genes. The study team conducted time-to-event analyses to estimate and compare percentiles in time with milestone attainment across autistic individuals, subgroups of autistic individuals, and the sibling sample.

**Results:**

Seventeen thousand ninety-eight autistic individuals (mean age, 9.15 years; 80.8% male) compared with 4145 siblings without autism or ID (mean age, 10.2 years; 50.2% female) showed delays in milestone attainment, with median (IQR) delays ranging from 0.7 (0.3-1.6) to 19.7 (11.4-32.2) months. More severe and more variable delays in autism were associated with the presence of co-occurring ID, carrying an NDD-associated rare genetic variant, and being diagnosed with autism by age 5 years. More severe and more variable delays were also associated with membership in earlier study cohorts, consistent with autism’s diagnostic and ascertainment expansion over the last 30 years.

**Conclusions and Relevance:**

As the largest summary to date of developmental milestone attainment in autism, to our knowledge, this study demonstrates substantial developmental variability across different conditions and provides important context for understanding the phenotypic and etiological heterogeneity of autism.

## Key Points

**Question:**

When do autistic individuals, on average, attain key developmental milestones?

**Findings:**

Using retrospective, parent-reported data from 17 098 autistic individuals in this cross-sectional study, a found substantial variability in average developmental milestone acquisition was found. Average delays increased with co-occurring intellectual disability, presence of a genetic variant associated with neurodevelopmental disorders, earlier autism diagnosis, and participation in older autism cohorts, and average delays also were larger for later milestones (eg, phrase speech, bowel control) than earlier milestones (eg, smiling, sitting).

**Meaning:**

These findings show that developmental milestone progress in autism varies substantially under different conditions, including presence of intellectual disability, genetic testing results, diagnosis timing, and study cohort membership.

## Teaser

This study characterizes variability in the age at which autistic individuals attain key developmental milestones compared with siblings who do not have an autism diagnosis.

## Introduction

Delays in milestone attainment (eg, delays in first words or phrases) are often the first signs of developmental differences in children who are later diagnosed with autism.^1^ The extensive variability in developmental progression preceding an autism diagnosis^2,3^ suggests that developmental milestone timing may capture an important facet of autism heterogeneity.^4^ While many reports have provided estimates of milestone timing averages (central tendency), few have characterized the full range of variability in milestone attainment across multiple developmental domains.^5,6,7,8^ By aggregating several large autism cohorts, we increase previous sample sizes by approximately an order of magnitude. In this study, we aimed to estimate developmental milestone averages and distributions in more than 17 000 autistic individuals, as well as in more than 4000 siblings of autistic children who do not have an autism diagnosis themselves. We further characterized milestone progression in several subgroups of autistic individuals: autistic male individuals and autistic female individuals, autistic individuals with co-occurring intellectual disability (ID), autistic individuals with a genetic variant associated with neurodevelopmental disorders (NDD), autistic individuals diagnosed early (before age 5 years), and autistic individuals diagnosed late (after age 10 years). We also examined milestone attainment differences by research cohort.

Many of these groups of autistic individuals have elevated rates of co-occurring ID. Co-occurring ID is associated with global developmental delays in and outside of autism, and is present in approximately 17% to 39% of autistic individuals in the US.^9^ It is more common in autistic individuals who carry disruptive, typically de novo, genetic variants in genes associated with NDD.^10,11,12^ ID is also more common in female individuals diagnosed with autism,^13,14^ which could reflect sex differences in both ascertainment and autism presentation.^15,16^ As ID, with or without autism, is associated with delays in attaining key milestones, groups of autistic individuals with higher rates of co-occurring ID often attain milestones later.^12,17,18^ The large amount of data aggregated here permits full characterization, and comparison, of milestone attainment across these groups of autistic individuals with ID.

Most averages in autism presentation are sensitive to time and place of diagnosis and evolve with ascertainment trends. On average, over the past decade and across 40 countries, children are diagnosed with autism around age 5 years.^19^ Autistic individuals diagnosed earlier in the US are more likely to have co-occurring ID or global developmental delay.^20^ However, even as rates of ID in autism have declined,^21,22^ autistic individuals are being diagnosed earlier on average than in previous years.^23,24^ Thus, controlling for age at study entry, recent cohorts may include higher proportions of autistic individuals who show milder developmental delays. To our knowledge, no studies to date have compared milestone attainment across autism cohorts ascertained across time. Here, we explore these associations across cohorts ascertained at different start dates over 25 years (1997 to present) to clarify the scope and stability of developmental heterogeneity in autism that is being captured in prominent research cohorts.

## Methods

### Sample

We aggregated data from 17 098 autistic individuals from 4 multisite cohorts (eTable 1 in the Supplement): the Autism Genetic Resource Exchange (AGRE; n = 3284),^25,26^ the Autism Simplex Collection (TASC; n = 694),^27^ the Simons Simplex Collection (SSC; n = 2753),^28^ and the Simons Foundation Powering Autism Research for Knowledge (SPARK; n = 10 367).^29^ The SPARK cohort also collected milestone data from siblings without an autism diagnosis; we therefore included 4145 siblings without autism or ID from the SPARK cohort as a comparison group. Autistic individuals and siblings without an autism diagnosis were included if they had a measured value for at least 1 of the developmental milestone variables (delineated below) and were between the ages of 4 and 17 years (as those ages were included in each of the 4 cohorts). Furthermore, 5295 autistic participants (30.8% of the autistic sample) had available exome sequencing data (including 43 from AGRE, 2048 from SSC, and 3204 from SPARK). Given that the milestones we aimed to characterize are, on average, attained in the general population by 4 years of age,^30^ the participants had aged past the normative age period for attaining the milestones of interest. The final sample included autistic participants who were on average 9.15 years old and siblings who were on average 10.20 years old (Table). Autistic participants were also 80% male, similar to population-level sex ratios for autism,^31^ whereas male and female siblings were evenly represented. Written informed consent and assent was provided for all participants in each cohort. The research specific to this study was approved by the Partners Healthcare Institutional Review Board and was reported following Strengthening the Reporting of Observational Studies in Epidemiology (STROBE) reporting guidelines.^32^

**Table.** Sample Characteristics

Characteristic	Autistic individuals (n = 17 098), No. (%)	Siblings without an autism diagnosis (n = 4145), No. (%)
Age, mean (SD), y	9.15 (3.6)	10.2 (3.81)
Sex		
Male	13 816 (80.8)	2065 (49.8)
Female	3282 (19.2)	2080 (50.2)
Co-occurring intellectual disability	9691	NA
Intellectual disability	3665 (37.8)	
No intellectual disability	6026 (62.2)	
Genetic etiology	5295	
Associated rare variant		
With NDD–associated rare variant	352 (6.6)	
Without NDD–associated rare variant	4943 (93.4)	
Age at autism diagnosis, y	10 367	
By age 5	6859 (66.2)	
At ages 5-9	2797 (27.0)	
After age 10	711 (6.9)	

Abbreviations: NA, not applicable; NDD, neurodevelopmental disorders.

### Procedures

At the time the participants were enrolled in each of the above studies, parents retrospectively reported the age at which participants attained the following developmental milestones (1) smiling, (2) gross motor skills (including sitting upright, crawling, and walking), (3) self-help skills (spoon-feeding self), (4) expressive language skills (speaking words and phrases), and (5) toileting (acquiring bladder control and acquiring bowel control). Age at milestone attainment was coded in months. Different milestones had different sample sizes as not all milestones were assessed by each study cohort. A full description of the sample size and assessment approach for each milestone can be found in eTable 2 in the Supplement. Analyses for each milestone were independent and not affected by differing sample sizes across milestones.

In the SSC, AGRE, and TASC cohorts, ID was defined as full-scale or nonverbal IQ score under 70. SPARK participants were also classified as having ID if they were reported by parents to have an estimated IQ score under 70, ID, significant cognitive impairment, global developmental delay, or borderline intellectual functioning. Presence of an NDD-associated genetic variant was defined to include de novo protein truncating variants, copy number variants, and missense variants with a Missense PolyPhen Constraint score^33^ greater than 2 (indicating that it is highly disruptive, on par with protein truncating variants) in 1 of 373 genes associated with NDD at an approximately genome-wide significant level through a recent exome sequencing study.^34^

### Statistical Analyses

Using observation-level data from each cohort, we modeled milestone attainment using a time-to-event approach with interval-censored outcomes,^35^ including (1) exact time-to-event, where the milestone was attained at a known age, coded [*t, t)*; (2) right-censored data, where the milestone was not yet attained by the age at assessment, *t*, coded [*t,* +∞); (3) left-censored data, where the milestone was attained by the age of assessment, *t*, but the age at attainment was not known, coded (–∞, *t*]; and (4) interval-censored data, where the milestone was attained after a specific age, *t*1, before the age at assessment, *t*2, coded (*t*1, *t*2). Time-to-event analysis methods are based on survival analysis methods; similar to how survival analyses estimate the time to a loss event in a population, time-to-event analyses estimate the time to a gain event population. We fitted parametric models in the overall autism sample based on Weibull, gamma, and log-normal distributions, and selected the model with the lowest Akaike information criterion (AIC).^36,37^ Given that the AIC was consistently lowest for the log-normal distribution across milestones, we selected the log-normal hazard function for all subsequent analyses.

We fitted time-to-event models in the overall autistic sample and the sibling sample. We then conducted time-to-event models by autistic subgroups based on (1) sex and ID, (2) age at diagnosis, (3) genetic findings, and (4) study cohort. We conducted these analyses 1 subgroup variable at a time, such that the sample sizes vary between analyses in a way that reflects which cohort(s) the subgroup variable was measured in. We used these time-to-event models to identify the 5th, 25th, 45th, 50th (median), 55th, 75th, and 95th percentiles of time to milestone attainment, equivalent to the ages at which different proportions of the sample (ie, 5%, 25%, etc) attained each milestone.

We conducted conservative 2-sided log-rank 2-sample permutation tests (with 100 permutations) to test pairwise differences between subgroup time-to-event distributions.^38,39^ We used false discovery rate (FDR)^40^ to correct for multiple comparisons in each set of analyses (eg, for the sex and ID analysis, given 4 subgroups of autistic individuals and 1 sibling subgroup, we adjusted *P* values for 10 pairwise subgroup comparisons per milestone, across 9 milestones, for a total of 90 pairwise comparisons). FDR-corrected *P* values were considered significant at *P* < .05.

## Results

### Overall Comparisons With Siblings

Compared with 4145 siblings without an autism diagnosis or ID (mean age, 10.2 years; 50.2% female), 17 098 autistic individuals (mean age, 9.15 years; 80.8% male) showed delays in milestone attainment, with median (IQR) delays ranging from 0.7 (0.3-1.6) to 19.7 (11.4-32.2) months. As expected, autistic individuals showed substantial delays in attaining milestones compared with their siblings without an autism diagnosis or ID, whose milestone attainment was generally consistent with and sometimes slightly earlier than general population norms.^41,42,43^ Of the autistic participants who provided data for phrase speech, bladder control, and bowel control, 502 participants (4.2%) had not attained any of these milestones. eTable 3 in the Supplement details percentiles for milestone attainment in autistic individuals, subgroups of autistic individuals, siblings without an autism diagnosis or ID, and a comparison set of general population estimates.^41,42,43^ eTable 4 in the Supplement presents pairwise comparisons of subgroup time-to-event distributions.

### Cohort

**Figure 1.**
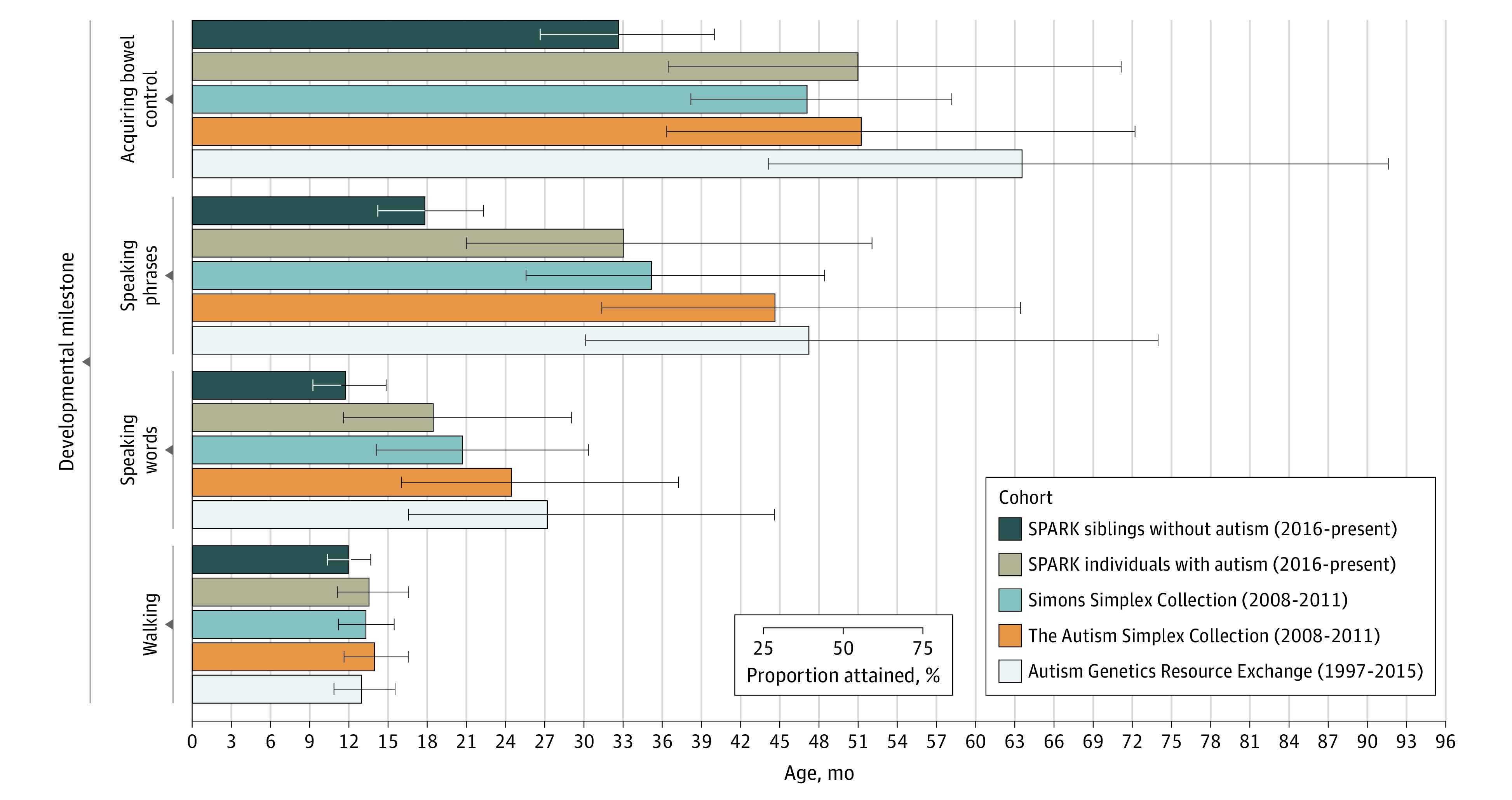
Developmental Milestones Attained by Autistic Individuals, Grouped by Cohort SPARK siblings without an autism diagnosis (n = 4145); SPARK autistic individuals (n = 10 367); Simons Simplex Collection (n = 2753); The Autism Simplex Collection (n = 694); and Autism Genetics Resource Exchange (n = 3284). See eTable 3 in the Supplement for milestone attainment percentiles and eTable 4 in the Supplement for pairwise subgroup comparisons of survival distributions.

Autistic individuals were ascertained from as early as 1997 in the AGRE cohort, 2008 in the TASC and SSC cohorts, and 2016 in the SPARK cohort. Compared with autistic individuals from the most recent cohort, autistic individuals from earlier cohorts showed greater and more variable delays for many milestones, including speaking words, speaking phrases, and acquiring bowel control (Figure 1 and eTable 5 in the Supplement). Specifically, all time-to-event distributions differed significantly, following multiple testing correction, between cohorts except between AGRE vs SSC and TASC vs SPARK for walking, SSC vs SPARK for speaking phrases, and TASC vs SPARK for acquiring bladder control. All significant pairwise cohort comparisons passed FDR-corrected *P* < .01, as detailed in eTable 6 in the Supplement.

### Co-occurring ID

**Figure 2.**
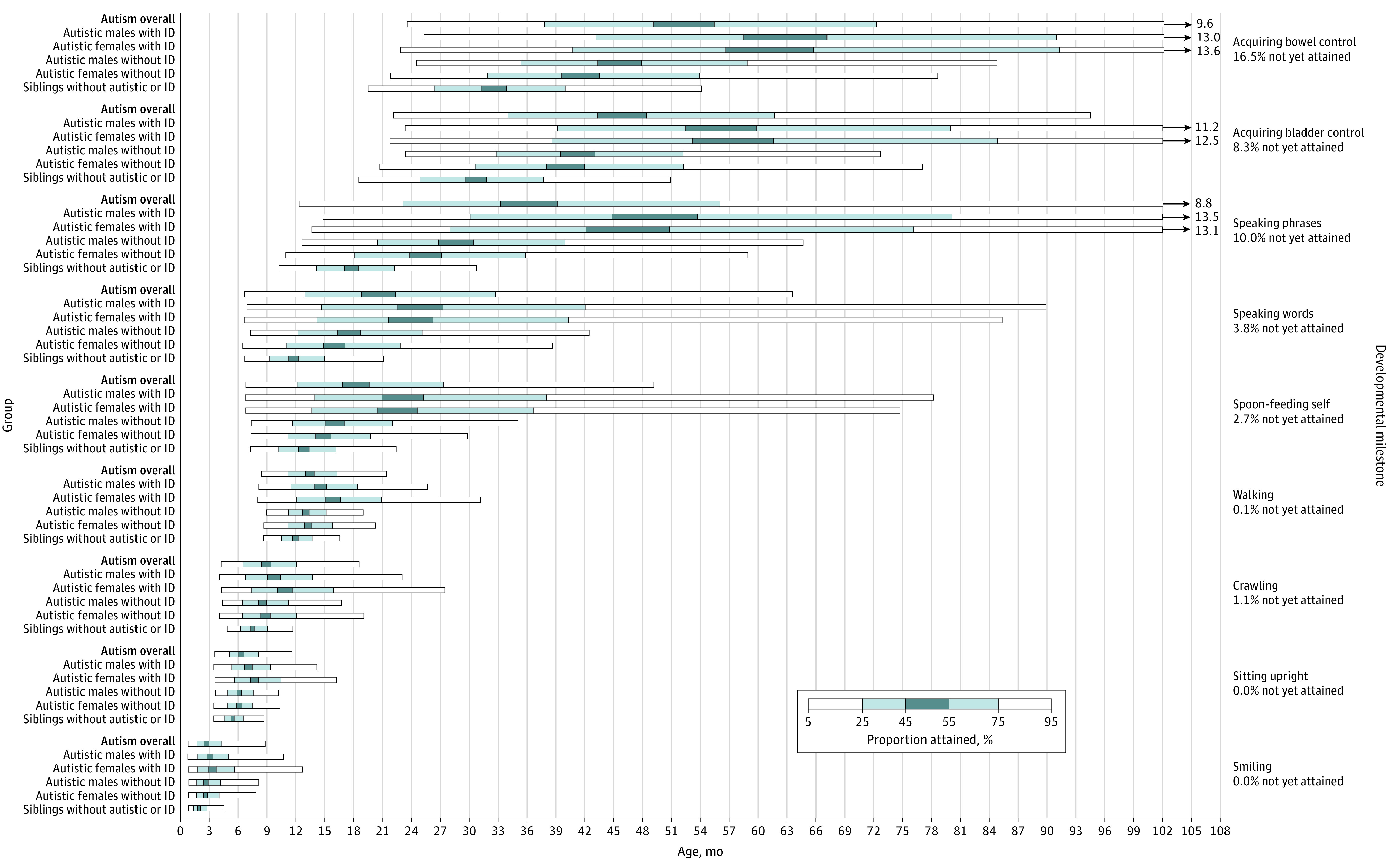
Developmental Milestones Attained by Autistic Individuals, Grouped by Sex and Intellectual Disability (ID) Developmental milestones not yet attained for overall autism sample. Siblings without an autism diagnosis or ID (n = 4145); autistic males with ID (n = 2875); autistic females with ID (n = 790); autistic males without ID (n = 5000); autistic females without ID (n = 1026); and autistic individuals overall (n = 17 098). Siblings without an autism diagnosis or ID show comparable developmental milestone attainment compared with the general population.^41,42,43^ See eTable 7 in the Supplement for milestone attainment percentiles and eTable 8 in the Supplement for pairwise subgroup comparisons of survival distributions.

As anticipated, autistic individuals with ID showed substantial additional delays in milestone attainment (Figure 2; and eTable 7 in the Supplement). We observed a gradient of severity and variability in developmental delays based on sex and co-occurring ID, with male individuals with ID showing the largest and most variable delays, followed by female individuals with ID, then male individuals without ID, then female individuals without ID, then siblings (eTable 8 in the Supplement). These gaps were already noticeable for the most early, basic milestones, and widened further as individuals progressed to later milestones. At the median ages for attaining milestones, these delays spanned 1 to 2 years for expressive language skills, such as speaking words and speaking phrases, and for toileting skills, such as acquiring bladder and acquiring bowel control. Notably, for individuals with co-occurring ID, the 95th percentile extended beyond age 11 years, or with a delay of more than 7 years, for speaking phrases, acquiring bladder control, and acquiring bowel control.

The study team further observed sex differences in individuals with ID (eTable 8 in the Supplement) but not in individuals without ID for earlier milestones (ie, smiling, sitting upright, crawling, and acquiring bladder control). In contrast, we observed the opposite trend for later milestones (ie, spoon-feeding self, speaking words, speaking phrases, acquiring bladder control, and acquiring bowel control), finding sex differences in individuals without ID but not in individuals with ID.

### Genetic Etiology

**Figure 3.**
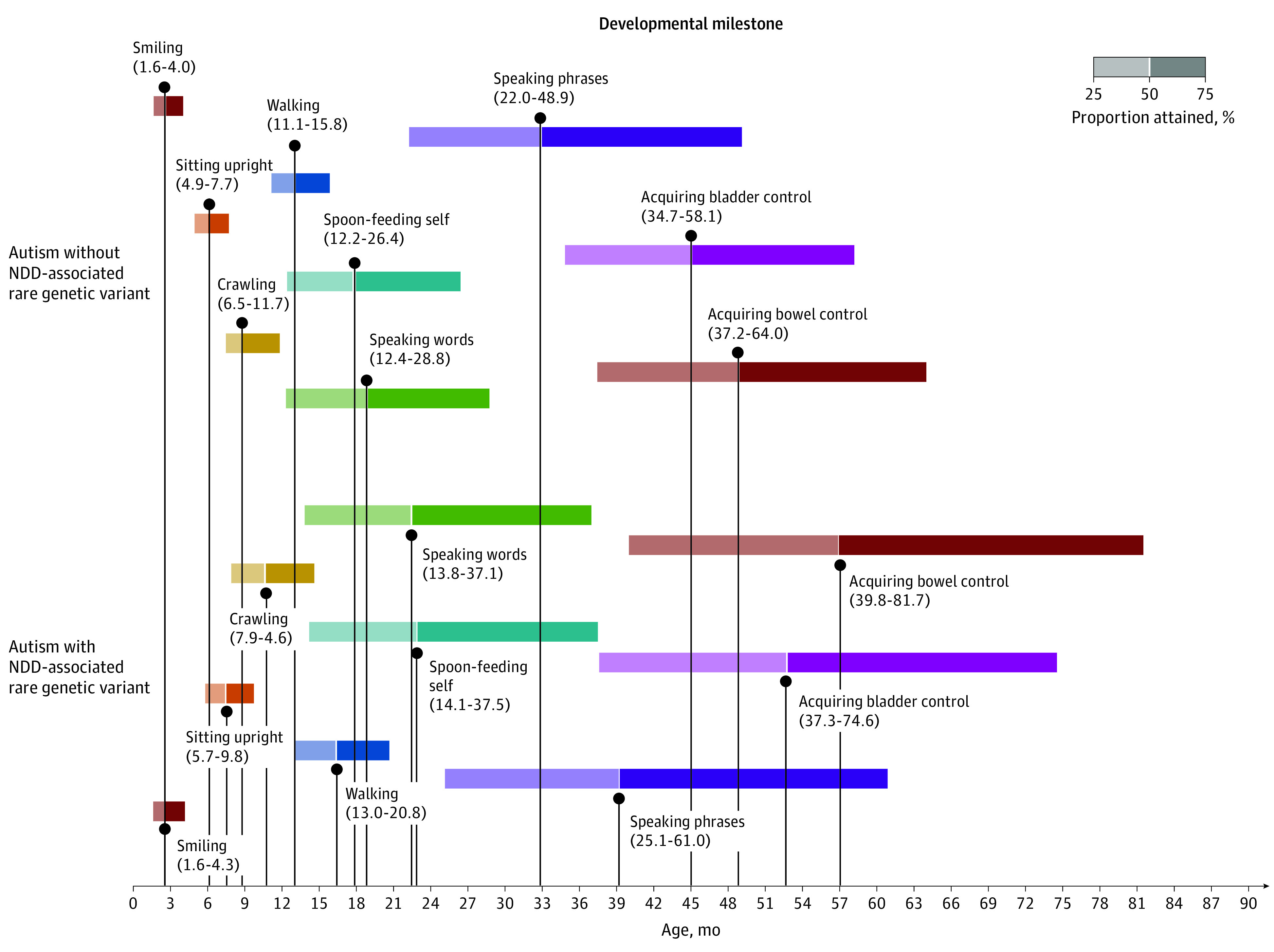
Developmental Milestones Attained by Autistic Individuals, Grouped by Genetic Etiology Autism without neurodevelopmental disorder (NDD)–associated rare genetic variant (n = 4943); and autistic with NDD-associated rare genetic variant (n = 352). Participants from AGRE, SPARK, and Simons Simplex Collection cohorts.. See eTable 9 in the Supplement for milestone attainment percentiles and eTable 10 in the Supplement for pairwise subgroup comparisons of survival distributions.

Approximately 352 of the genetically characterized autistic individuals (6.6%) harbored a genetic variant that arose de novo and disrupted the coding sequence of an NDD-associated gene. Carrying an NDD-associated variant substantially increased delays across all milestones (Figure 3 and eTable 9 in the Supplement) except for smiling, for which autistic individuals with a known variant did not differ from those without a known rare variant (eTable 10 in the Supplement).

### Diagnosis Timing

**Figure 4.**
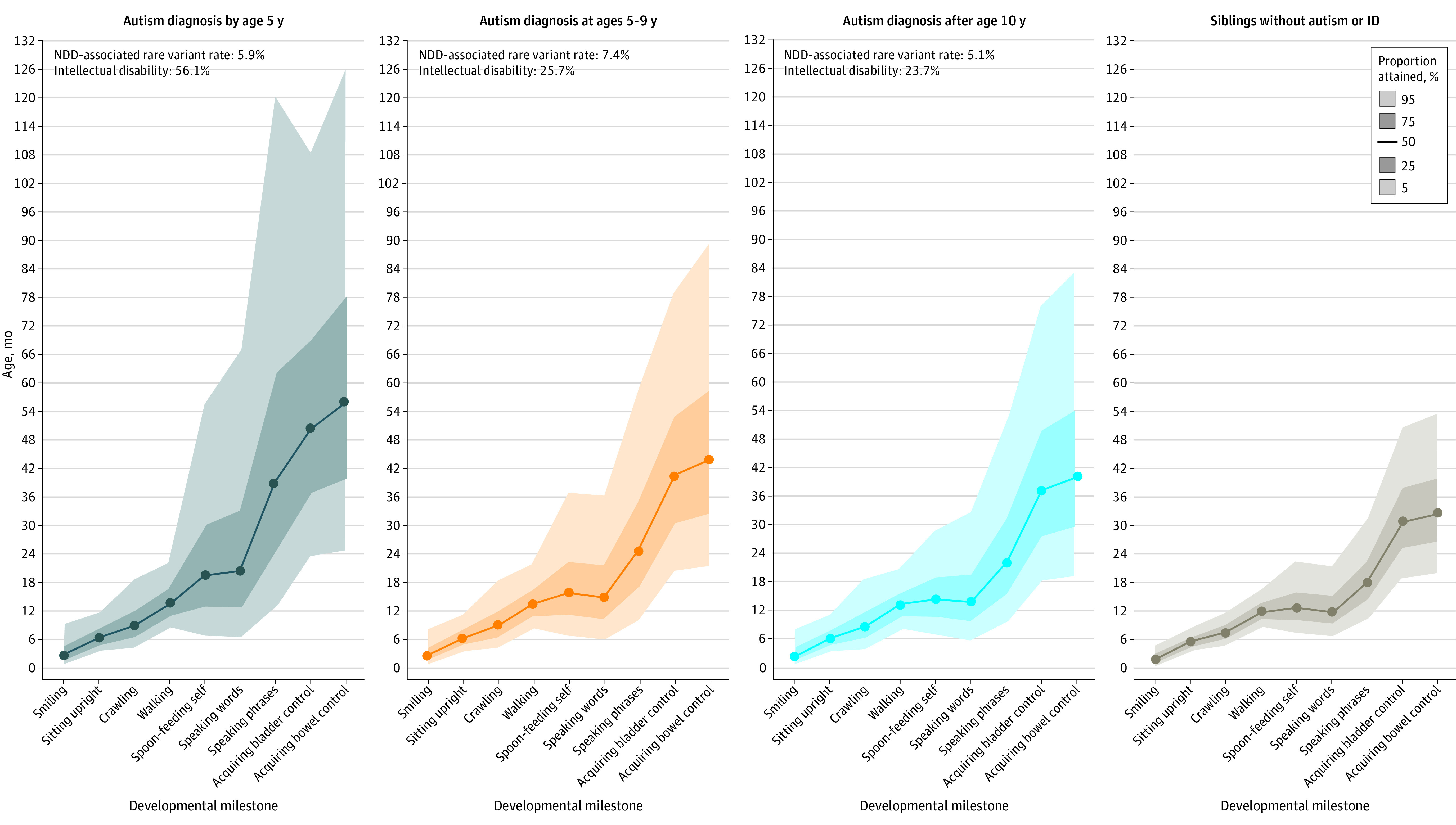
Developmental Milestones Attained by Autistic Individuals, Grouped by Age at Autism Diagnosis Autism diagnosed by age 5 years (n = 6859); autism diagnosed at age 5 to 9 years (n = 2797); autism diagnosed after age 10 years (n = 711); and siblings without an autism diagnosis or intellectual disability (ID) (n = 4145). Participants from SPARK cohort. See eTable 11 in the Supplement for milestone attainment percentiles and eTable 12 in the Supplement for pairwise subgroup comparisons of survival distributions. NDD indicates neurodevelopmental disorders.

Developmental delays were most substantial for autistic individuals diagnosed at or before age 5 years, followed by autistic individuals diagnosed at ages 5 to 9 years, then by autistic individuals diagnosed after age 10 years (Figure 4, eTable 11, and eTable 12 in the Supplement). Autistic children diagnosed by age 5 years were significantly more likely to have co-occurring ID than autistic children diagnosed at ages 5 to 9 years (56.1% vs 23.7%; χ^2^_1_ = 428.998; *P* < .001) or autistic children diagnosed after age 10 years (56.1% vs 25.7%; χ^2^_1_ = 172.985; *P* < .001). The latter 2 groups did not differ significantly from each other (23.7% vs 25.7%; χ^2^_1_ = 0.675; *P* = .41). We found no differences between those diagnosed at ages 5 to 9 years and those diagnosed after age 10 years for early milestones including smiling, sitting upright, and crawling. Furthermore, for crawling and walking, cases diagnosed by age 5 years did not differ significantly from those diagnosed at ages 5 to 9 years.

## Discussion

Early milestone attainment is highly variable among autistic individuals. More severe and more variable delays in autism were associated with the presence of co-occurring ID, carrying an NDD-associated genetic variant, and being diagnosed with autism by age 5 years. More severe and more variable delays were also associated with membership in earlier study cohorts, consistent with autism’s diagnostic and ascertainment expansion over the last 30 years. In light of the observed cohort effects, our findings should be taken in the context of constantly evolving conceptualizations of how core autism symptoms manifest. Acknowledging that no single study to date, no matter how large, can be fully or stably representative of autism, our findings highlight the extent to which estimates of milestone attainment, and many other phenotypic averages in autism, are influenced by diagnosis timing and ascertainment.^44,45,46,47^

Co-occurring ID substantially extends the timeline of developmental milestone attainment in autism. Age at walking was an exception, however, as autistic individuals with and without ID did not differ in this milestone. These findings build upon previous work suggesting that for some autistic children (ie, many of whom acquire early milestones as expected), ID may be a secondary consequence of the autism. Thus, differences in attainment of basic milestones like age at walking may not differentiate between autistic individuals with or without ID to the same extent within autism cohorts (compared with non–autism cohorts), and are more likely to signal the presence of a rare genetic variant in autism.^6,48,49^ The unprecedentedly large scale of this study also enabled us to examine developmental variability in individuals who carry an NDD-associated rare genetic variant. We used a strict threshold to define genetic variants. Given that most genetic variants are not captured in this category (such as common variants that each contribute less to overall likelihood of an autism diagnosis than rare variants),^50,51^ future research will be able to consider a broader set of genetic variants.

Our study bears several advantages over prior studies. We harmonized 4 cohorts to evaluate multiple developmental milestones across diverse behavioral domains, and included a large comparison sample of siblings without an autism diagnosis or ID. Participants were sampled from multiple developmental periods spanning childhood to late adolescence, enabling us to measure a wide range of possible ages for attaining developmental milestones. Given that population health registries to date have recorded relatively limited information on developmental milestones, this study is the most comprehensive analysis at this scale of developmental milestone attainment in autism.

### Limitations

Our findings should be considered in light of certain limitations. Variability in developmental milestone attainment is likely attributable not only to autism but to contextual factors, such as cultural practices, particularly given that most of the sample was based in the US. Our measures of developmental progress were retrospective and based on parent recall and would be bolstered by converging evidence from longitudinal measures, such as prospective clinician records. Siblings without an autism diagnosis showed slightly earlier ages at attainment for some milestones compared with the general population, which may reflect differences between children without an autism diagnosis ascertained based on having an autistic sibling vs children without an autism diagnosis ascertained in the general population. Similarly, siblings without an autism diagnosis were from 1 cohort and may not be representative of siblings without an autism diagnosis from other cohorts. The cohort effects suggest that the autism sample is not, and cannot be, representative of all autistic individuals. This limitation is not unique to our study design and remains challenging for all autism studies.

## Conclusion

As the largest summary to date of developmental milestone attainment in autism, this study highlights the extensive developmental variability within and across different contexts of autism. Our work emphasizes the utility of capturing not only average developmental progress, but also variability in developmental progress, to elucidate the causes and courses of autism.

## Notes

Supplement. eTable 1. Cohort characteristics. eTable 2. Sample size and assessment approach for each developmental milestone. eTable 3. Percentiles of months to milestone attainment in overall autism sample and sibling sample. eTable 4. Comparisons of months to milestone attainment between overall autism sample and sibling sample. eTable 5. Percentiles of months to milestone attainment grouped by cohort. eTable 6. Comparisons of months to milestone attainment grouped by cohort. eTable 7. Percentiles of months to milestone attainment grouped by sex and intellectual disability. eTable 8. Comparisons of months to milestone attainment grouped by sex and intellectual disability. eTable 9. Percentiles of months to milestone attainment grouped by genetic etiology. eTable 10. Comparisons of months to milestone attainment grouped by genetic etiology. eTable 11. Percentiles of months to milestone attainment grouped by age at autism diagnosis. eTable 12. Comparisons of months to milestone attainment grouped by age at autism diagnosis. eReferences.
