# Supplementary material for: Developmental Variability in Autism Across 17 000 Autistic Individuals and 4000 Siblings Without an Autism Diagnosis: Comparisons by Cohort, Intellectual Disability, Genetic Etiology, and Age at Diagnosis
Source: JAMA Pediatr. 2022 Jul 18;176(9):915–23. doi: 10.1001/jamapediatrics.2022.2423 (PMC9295026; doi:10.1001/jamapediatrics.2022.2423)
Supplement: Supplement. — eTable 1. Cohort characteristics. eTable 2. Sample size and assessment approach for each developmental milestone. eTable 3. Percentiles of months to milestone attainment in overall autism sample and sibling sample. eTable 4. Comparisons of months to milestone attainment between overall autism sample and sibling sample. eTable 5. Percentiles of months to milestone attainment grouped by cohort. eTable 6. Comparisons of months to milestone attainment grouped by cohort. eTable 7. Percentiles of months to milestone attainment grouped by sex and intellectual disability. eTable 8. Comparisons of months to milestone attainment grouped by sex and intellectual disability. eTable 9. Percentiles of months to milestone attainment grouped by genetic etiology. eTable 10. Comparisons of months to milestone attainment grouped by genetic etiology. eTable 11. Percentiles of months to milestone attainment grouped by age at autism diagnosis. eTable 12. Comparisons of months to milestone attainment grouped by age at autism diagnosis. eReferences. [file jamapediatr-e222423-s001.pdf]

Supplemental Online Content

Kuo SS, van der Merwe C, Fu JM, et al. Developmental variability in autism across 17 000 autistic individuals and 4000 siblings without an autism diagnosis: comparisons by cohort, intellectual disability, genetic etiology, and age at diagnosis. *JAMA Pediatr*. Published online July 18, 2022. doi:10.1001/jamapediatrics.2022.2423

eTable 1. Cohort characteristics.
eTable 2. Sample size and assessment approach for each developmental milestone.
eTable 3. Percentiles of months to milestone attainment in overall autism sample and sibling sample.
eTable 4. Comparisons of months to milestone attainment between overall autism sample and sibling sample.
eTable 5. Percentiles of months to milestone attainment grouped by cohort.
eTable 6. Comparisons of months to milestone attainment grouped by cohort.
eTable 7. Percentiles of months to milestone attainment grouped by sex and intellectual disability.
eTable 8. Comparisons of months to milestone attainment grouped by sex and intellectual disability.
eTable 9. Percentiles of months to milestone attainment grouped by genetic etiology.
eTable 10. Comparisons of months to milestone attainment grouped by genetic etiology.
eTable 11. Percentiles of months to milestone attainment grouped by age at autism diagnosis.
eTable 12. Comparisons of months to milestone attainment grouped by age at autism diagnosis.
eReferences.

This supplemental material has been provided by the authors to give readers additional information about their work.

© 2022 Kuo SS et al. *JAMA Pediatrics*.

eTable 1. Cohort characteristics.

Cohort	*n*	Recruitment	Ascertainment	Exclusions
Autism Genetic Resource Exchange (AGRE)[1,2]	3,258	1997-2015, US	Largely multiplex families, aged 2-51, research diagnosis*	Known genetic or medical cause of autism
The Autism Simplex Collection (TASC)[3]	706	2008-2010, North America and Europe	Simplex families, aged 3-21, research diagnosis*	IQ<35 and/or a mental age<18 months, known medical or genetic cause of autism, or suspected childhood disintegrative disorder
Simons Simplex Collection (SSC)[4]	2,755	2008-2011, North America	Simplex families, aged 4-18, research diagnosis*	Mental age<18 months, severe neurological deficits, birth trauma, perinatal complications, or fragile X or Down syndromes
Simons Foundation Powering Autism Research for Knowledge (SPARK)[5]	10,432	2016-present, US	Simplex and multiple families, aged 1-85, reported professional diagnosis	

Note. *Research diagnosis of autism spectrum disorder or autism based on Autism Diagnostic Interview-Revised (ADI-R)[6] or the Autism Diagnostic Observation Schedule (ADOS)[7]

© 2022 Kuo SS et al. *JAMA Pediatrics*.

eTable 2. Sample size and assessment approach for each developmental milestone.

Milestone	*n* Cases	*n* Siblings	Assessment Approach
Smiling	10,148	4,094	Background History Questionnaire (SPARK): Age in months when first smiled
Sitting Upright	10,075	4,065	Background History Questionnaire (SPARK): Age in months when first sat without support
Crawling	10,012	4,056	Background History Questionnaire (SPARK): Age in months when first crawled
Walking	16,815	4,121	Background History Questionnaire (SPARK): Age in months when first walked alone; ADI-R (AGRE, TASC, SSC): Age when first walked unaided
Spoon-Feeding Self	9,919	3,968	Background History Questionnaire (SPARK): Age in months when first fed self with a spoon
Speaking First Word	16,523	3,931	Background History Questionnaire (SPARK): Age in months when first used single words; ADI-R (AGRE, TASC, SSC): Age of first single words
Speaking First Phrase	16,304	3,975	Background History Questionnaire (SPARK): Age in months when first combined words into short phrases or sentences with an action word; ADI-R (AGRE, TASC, SSC): Age of first phrases
Attaining Bladder Control	12,695	4,121	Background History Questionnaire (SPARK): Age in months when first was bladder-trained (months); ADI-R (AGRE, SSC): Acquisition of bladder control (months)
Attaining Bowel Control	15,974	4,112	Background History Questionnaire (SPARK): Age in months when first was bowel-trained; ADI-R (AGRE, TASC, SSC): Acquisition of bowel control

Note. SPARK: Simons Foundation Powering Autism Research Knowledge. AGRE: Autism Genetics Resource Exchange. SSC: Simons Simplex Collection. TASC: The Autism Simplex Collection. ADI-R: Autism Diagnostic Interview-Revised[6].

© 2022 Kuo SS et al. *JAMA Pediatrics*.

eTable 3. Percentiles of months to milestone attainment in overall autism sample and sibling sample.

Milestone	Group	*n*	Censored	% Censored	5%	25%	45%	50%	55%	75%	95%
Smiling	General population (b)							1		1	
Smiling	Siblings without an autism diagnosis or ID	4094	0	0.0	0.8	1.3	1.7	1.9	2.0	2.7	4.5
Smiling	Autistic individuals	10148	5	0.0	0.8	1.6	2.4	2.6	2.9	4.3	8.7
Sitting Upright	General population (c)				4	5		6		7	8
Sitting Upright	Siblings without an autism diagnosis or ID	4065	1	0.0	3.4	4.5	5.2	5.4	5.6	6.5	8.6
Sitting Upright	Autistic individuals	10075	2	0.0	3.5	5.0	6.0	6.3	6.6	8.1	11.5
Crawling	General population (c)				6	7		8		9	11
Crawling	Siblings without an autism diagnosis or ID	4056	6	0.1	4.8	6.2	7.2	7.5	7.7	9.0	11.6
Crawling	Autistic individuals	10012	111	1.1	4.2	6.5	8.4	8.8	9.4	12.0	18.5
Walking	General population (c)				9	11		12		13	15
Walking	Siblings without an autism diagnosis or ID	4121	4	0.1	0.1	10.4	11.6	11.9	12.2	13.6	16.5
Walking	Autistic individuals	16815	15	0.1	8.4	11.1	12.9	13.4	13.9	16.2	21.4
Spoon-Feeding Self	General population (b)					13		15		17	
Spoon-Feeding Self	Siblings without an autism diagnosis or ID	3968	5	0.1	7.2	10.1	12.2	12.7	13.3	16.1	22.4
Spoon-Feeding Self	Autistic individuals	9919	267	2.7	6.7	12.1	16.8	18.2	19.6	27.3	49.1
Speaking Word	General population (b)					10		12		14	
Speaking Word	Siblings without an autism diagnosis or ID	3931	3	0.1	6.6	9.2	11.2	11.7	12.3	14.9	21.0
Speaking Word	Autistic individuals	16523	634	3.8	6.6	12.9	18.8	20.5	22.3	32.6	63.5
Speaking Phrase	General population (b)					17		20		23	
Speaking Phrase	Siblings without an autism diagnosis or ID	3975	8	0.2	10.2	14.1	17.0	17.7	18.5	22.2	30.7
Speaking Phrase	Autistic individuals	16304	1629	10.0	12.2	23.1	33.2	36.0	39.1	56.0	105.9
Acquiring Bladder Control	General population (a)					28		34		38	
Acquiring Bladder Control	Siblings without an autism diagnosis or ID	4121	23	0.6	18.5	24.9	29.5	30.6	31.8	37.7	50.8
Acquiring Bladder Control	Autistic individuals	12695	1058	8.3	22.1	34.0	43.3	45.8	48.4	61.6	94.5
Acquiring Bowel Control	General population (a)					28		34		38	
Acquiring Bowel Control	Siblings without an autism diagnosis or ID	4112	35	0.9	19.5	26.3	31.2	32.5	33.8	40.0	54.1
Acquiring Bowel Control	Autistic individuals	15974	2628	16.5	23.6	37.7	49.1	52.2	55.4	72.2	115.4

Note. ID: Intellectual disability. General population norms adapted from[8-10]:
(a) Schum TR, Kolb TM, McAuliffe TL, Simms MD, Underhill RL, Lewis M. Sequential acquisition of toilet-training skills: A descriptive study of gender and age differences in normal children. *Pediatrics.* 2002;109(3):e48-e48. doi:10.1542/peds.109.3.e48
(b) Frankenburg WK, Dodds J, Archer P, Shapiro H, Bresnick B. The Denver II: A major revision and restandardization of the Denver Developmental Screening Test. *Pediatrics.* 1992;89(1):91-7.
(c) WHO Multicentre Growth Reference Study Group. WHO Motor Development Study: Windows of achievement for six gross motor development milestones. *Acta Paediatr Suppl.* 2006;450:86-95. doi:10.1111/j.1651-2227.2006.tb02379.x

© 2022 Kuo SS et al. *JAMA Pediatrics*.

eTable 4. Comparisons of months to milestone attainment between overall autism sample and sibling sample.

Milestone	Comparison	χ2	*df*	Adjusted *p*
Smiling	Siblings without an autism diagnosis or ID / Autistic individuals	26.743	1	<0.001*
Sitting Upright	Siblings without an autism diagnosis or ID / Autistic individuals	24.955	1	<0.001*
Crawling	Siblings without an autism diagnosis or ID / Autistic individuals	25.114	1	<0.001*
Walking	Siblings without an autism diagnosis or ID / Autistic individuals	26.274	1	<0.001*
Spoon-Feeding Self	Siblings without an autism diagnosis or ID / Autistic individuals	33.244	1	<0.001*
Speaking Word	Siblings without an autism diagnosis or ID / Autistic individuals	42.254	1	<0.001*
Speaking Phrase	Siblings without an autism diagnosis or ID / Autistic individuals	51.342	1	<0.001*
Acquiring Bladder Control	Siblings without an autism diagnosis or ID / Autistic individuals	46.421	1	<0.001*
Acquiring Bowel Control	Siblings without an autism diagnosis or ID / Autistic individuals	50.964	1	<0.001*

Note. Asterisk denotes significant FDR-adjusted *p*-value<0.05. ID: Intellectual disability.

© 2022 Kuo SS et al. *JAMA Pediatrics*.

eTable 5. Percentiles of months to milestone attainment grouped by cohort.

Milestone	Group	*n*	Censored	% Censored	5%	25%	45%	50%	55%	75%	95%
Walking	Autism Genetics Resource Exchange (1997-2015)	3150	4	0.1	8.4	10.8	12.5	12.9	13.4	15.5	20.1
Walking	The Autism Simplex Collection (2008-2011)	682	0	0.0	9.0	11.6	13.4	13.9	14.4	16.6	21.3
Walking	Simons Simplex Collection (2008-2011)	2735	0	0.0	8.9	11.2	12.8	13.1	13.5	15.4	19.4
Walking	SPARK Autistic individuals (2016-Present)	10248	11	0.1	8.3	11.1	13.1	13.6	14.1	16.6	22.2
Walking	SPARK Siblings without an autism diagnosis (2016-Present)	4121	4	0.1	8.6	10.4	11.6	11.9	12.2	13.6	16.5
Speaking Word	Autism Genetics Resource Exchange (1997-2015)	3273	261	8.0	8.2	16.6	24.8	27.2	29.8	44.5	90.2
Speaking Word	The Autism Simplex Collection (2008-2011)	678	28	4.1	8.7	16.0	22.6	24.4	26.5	37.3	68.7
Speaking Word	Simons Simplex Collection (2008-2011)	2648	0	0.0	8.2	14.2	19.3	20.8	22.3	30.4	52.5
Speaking Word	SPARK Autistic individuals (2016-Present)	9924	345	3.5	6.0	11.6	16.9	18.4	20.0	29.1	56.5
Speaking Word	SPARK Siblings without an autism diagnosis (2016-Present)	3931	3	0.1	6.6	9.2	11.2	11.7	12.3	14.9	21.0
Speaking Phrase	Autism Genetics Resource Exchange (1997-2015)	3248	646	19.9	15.7	30.0	43.3	47.1	51.2	73.8	141.0
Speaking Phrase	The Autism Simplex Collection (2008-2011)	663	103	15.5	18.7	31.2	41.6	44.5	47.5	63.4	105.5
Speaking Phrase	Simons Simplex Collection (2008-2011)	2478	0	0.0	16.1	25.5	33.1	35.2	37.4	48.5	77.0
Speaking Phrase	SPARK Autistic individuals (2016-Present)	9915	880	8.9	10.7	20.8	30.2	32.9	35.8	52.0	100.5
Speaking Phrase	SPARK Siblings without an autism diagnosis (2016-Present)	3975	8	0.2	10.2	14.1	17.0	17.7	18.5	22.2	30.7
Acquiring Bowel Control	Autism Genetics Resource Exchange (1997-2015)	3143	1121	35.7	26.1	44.2	59.6	63.8	68.4	92.1	156.1
Acquiring Bowel Control	The Autism Simplex Collection (2008-2011)	644	156	24.2	22.3	36.3	47.8	50.9	54.3	71.5	116.3
Acquiring Bowel Control	Simons Simplex Collection (2008-2011)	2068	1	0.0	28.0	38.2	45.6	47.5	49.4	58.9	80.4
Acquiring Bowel Control	SPARK Autistic individuals (2016-Present)	10119	1350	13.3	22.5	36.3	47.6	50.7	53.9	70.6	114.0
Acquiring Bowel Control	SPARK Siblings without an autism diagnosis (2016-Present)	4112	35	0.9	19.5	26.3	31.2	32.5	33.8	40.0	54.1

Note. SPARK: Simons Foundation Powering Autism Research Knowledge. SPARK Siblings without an autism diagnosis (*n*=4,145); SPARK Autistic individuals (*n*=10,367); Simons Simplex Collection (*n*=2,753); The Autism Simplex Collection (*n*=694); Autism Genetics Resource Exchange (*n*=3,284).

© 2022 Kuo SS et al. *JAMA Pediatrics*.

eTable 6. Comparisons of months to milestone attainment grouped by cohort.

Milestone	Comparison	χ2	*df*	Adjusted *p*
Walking	SPARK Siblings / AGRE	17.345	1	<0.001*
Walking	SPARK Siblings / TASC	19.339	1	<0.001*
Walking	SPARK Siblings / SSC	19.273	1	<0.001*
Walking	SPARK Siblings / SPARK Autism	27.207	1	<0.001*
Walking	AGRE / TASC	6.216	1	<0.001*
Walking	AGRE / SSC	1.511	1	0.136
Walking	AGRE / SPARK Autism	8.284	1	<0.001*
Walking	TASC / SSC	5.353	1	<0.001*
Walking	TASC / SPARK Autism	1.553	1	0.128
Walking	SSC / SPARK Autism	6.388	1	<0.001*
Speaking Word	SPARK Siblings / AGRE	48.199	1	<0.001*
Speaking Word	SPARK Siblings / TASC	37.148	1	<0.001*
Speaking Word	SPARK Siblings / SSC	40.567	1	<0.001*
Speaking Word	SPARK Siblings / SPARK Autism	36.512	1	<0.001*
Speaking Word	AGRE / TASC	4.491	1	<0.001*
Speaking Word	AGRE / SSC	17.415	1	<0.001*
Speaking Word	AGRE / SPARK Autism	25.531	1	<0.001*
Speaking Word	TASC / SSC	7.192	1	<0.001*
Speaking Word	TASC / SPARK Autism	8.969	1	<0.001*
Speaking Word	SSC / SPARK Autism	4.456	1	<0.001*
Speaking Phrase	SPARK Siblings / AGRE	55.540	1	<0.001*
Speaking Phrase	SPARK Siblings / TASC	45.300	1	<0.001*
Speaking Phrase	SPARK Siblings / SSC	48.070	1	<0.001*
Speaking Phrase	SPARK Siblings / SPARK Autism	46.397	1	<0.001*

© 2022 Kuo SS et al. *JAMA Pediatrics*.

Speaking Phrase	AGRE / TASC	3.324	1	0.001*
Speaking Phrase	AGRE / SSC	21.472	1	<0.001*
Speaking Phrase	AGRE / SPARK Autism	23.734	1	<0.001*
Speaking Phrase	TASC / SSC	11.566	1	<0.001*
Speaking Phrase	TASC / SPARK Autism	9.472	1	<0.001*
Speaking Phrase	SSC / SPARK Autism	1.404	1	0.164
Acquiring Bladder Control	SPARK Siblings / AGRE	55.540	1	<0.001*
Acquiring Bladder Control	SPARK Siblings / TASC	28.927	1	<0.001*
Acquiring Bladder Control	SPARK Siblings / SSC	39.810	1	<0.001*
Acquiring Bladder Control	SPARK Siblings / SPARK Autism	45.881	1	<0.001*
Acquiring Bladder Control	AGRE / TASC	3.324	1	0.001*
Acquiring Bladder Control	AGRE / SSC	21.472	1	<0.001*
Acquiring Bladder Control	AGRE / SPARK Autism	23.734	1	<0.001*
Acquiring Bladder Control	TASC / SSC	7.379	1	<0.001*
Acquiring Bladder Control	TASC / SPARK Autism	2.615	1	0.010*
Acquiring Bladder Control	SSC / SPARK Autism	8.789	1	<0.001*
Acquiring Bowel Control	SPARK Siblings / AGRE	54.901	1	<0.001*
Acquiring Bowel Control	SPARK Siblings / TASC	31.865	1	<0.001*
Acquiring Bowel Control	SPARK Siblings / SSC	39.372	1	<0.001*
Acquiring Bowel Control	SPARK Siblings / SPARK Autism	47.531	1	<0.001*
Acquiring Bowel Control	AGRE / TASC	8.258	1	<0.001*
Acquiring Bowel Control	AGRE / SSC	25.081	1	<0.001*
Acquiring Bowel Control	AGRE / SPARK Autism	21.113	1	<0.001*
Acquiring Bowel Control	TASC / SSC	7.091	1	<0.001*
Acquiring Bowel Control	TASC / SPARK Autism	0.841	1	0.400
Acquiring Bowel Control	SSC / SPARK Autism	11.096	1	<0.001*

© 2022 Kuo SS et al. *JAMA Pediatrics*.

Note. Asterisk denotes significant FDR-adjusted p-value<0.05. SPARK Siblings (n=4,145); SPARK ASD (n=10,367); Simons Simplex Collection (n=2,753); The Autism Simplex Collection (n=694); Autism Genetics Resource Exchange (n=3,284).

© 2022 Kuo SS et al. *JAMA Pediatrics*.

eTable 7. Percentiles of months to milestone attainment grouped by sex and intellectual disability.

Milestone	Group	*n*	Censored	% Censored	5%	25%	45%	50%	55%	75%	95%
Smiling	Siblings without an autism diagnosis or ID	4094	0	0.0	0.8	1.3	1.7	1.9	2.0	2.7	4.5
Smiling	Autistic females without ID	592	0	0.0	0.8	1.6	2.3	2.5	2.8	4.0	7.8
Smiling	Autistic males without ID	2551	0	0.0	0.8	1.6	2.4	2.6	2.8	4.1	8.0
Smiling	Autistic females with ID	586	1	0.2	0.8	1.8	2.9	3.2	3.6	5.6	12.6
Smiling	Autistic males with ID	1925	3	0.2	0.8	1.7	2.7	3.0	3.3	5.0	10.7
Sitting Upright	Siblings without an autism diagnosis or ID	4065	1	0.0	3.4	4.5	5.2	5.4	5.6	6.5	8.6
Sitting Upright	Autistic females without ID	591	0	0.0	3.5	4.9	5.8	6.0	6.3	7.5	10.3
Sitting Upright	Autistic males without ID	2525	0	0.0	3.6	4.9	5.8	6.0	6.3	7.5	10.1
Sitting Upright	Autistic females with ID	587	0	0.0	3.6	5.6	7.2	7.6	8.1	10.4	16.1
Sitting Upright	Autistic males with ID	1906	2	0.1	3.5	5.3	6.6	7.0	7.4	9.3	14.1
Crawling	Siblings without an autism diagnosis or ID	4056	6	0.1	4.8	6.2	7.2	7.5	7.7	9.0	11.6
Crawling	Autistic females without ID	584	8	1.4	4.0	6.4	8.3	8.8	9.3	12.0	19.0
Crawling	Autistic males without ID	2514	25	1.0	4.3	6.4	8.1	8.5	8.9	11.2	16.7
Crawling	Autistic females with ID	576	12	2.1	4.2	7.3	10.0	10.8	11.6	15.8	27.4
Crawling	Autistic males with ID	1897	28	1.5	4.0	6.7	9.0	9.6	10.3	13.7	23.0
Walking	Siblings without an autism diagnosis or ID	4121	4	0.1	8.6	10.4	11.6	11.9	12.2	13.6	16.5
Walking	Autistic females without ID	1003	1	0.1	8.6	11.1	12.8	13.2	13.6	15.7	20.2
Walking	Autistic males without ID	4934	1	0.0	8.9	11.1	12.6	13.0	13.3	15.1	18.9
Walking	Autistic females with ID	777	2	0.3	8.0	12.0	15.0	15.8	16.6	20.9	31.1
Walking	Autistic males with ID	2833	8	0.3	8.1	11.4	13.8	14.4	15.1	18.3	25.6
Spoon-Feeding Self	Siblings without an autism diagnosis or ID	3968	5	0.1	7.2	10.1	12.2	12.7	13.3	16.1	22.4
Spoon-Feeding Self	Autistic females without ID	571	0	0.0	7.3	11.1	14.0	14.8	15.6	19.7	29.8
Spoon-Feeding Self	Autistic males without ID	2493	14	0.6	7.3	11.6	15.0	16.0	17.0	22.0	35.0
Spoon-Feeding Self	Autistic females with ID	581	35	6.0	6.7	13.6	20.4	22.4	24.5	36.6	74.7
Spoon-Feeding Self	Autistic males with ID	1893	121	6.4	6.7	13.9	20.9	23.0	25.2	38.0	78.2
Speaking Word	Siblings without an autism diagnosis or ID	3931	3	0.1	6.6	9.2	11.2	11.7	12.3	14.9	21.0
Speaking Word	Autistic females without ID	986	2	0.2	6.5	11.0	14.8	15.8	17.0	22.8	38.6
Speaking Word	Autistic males without ID	4840	17	0.4	7.2	12.1	16.3	17.4	18.7	25.1	42.4
Speaking Word	Autistic females with ID	762	53	7.0	6.6	14.1	21.6	23.8	26.2	40.2	85.4
Speaking Word	Autistic males with ID	2743	183	6.7	6.8	14.6	22.5	24.8	27.3	42.0	89.9
Speaking Phrase	Siblings without an autism diagnosis or ID	3975	8	0.2	10.2	14.1	17.0	17.7	18.5	22.2	30.7
Speaking Phrase	Autistic females without ID	982	9	0.9	10.9	18.0	23.8	25.4	27.1	35.8	58.9
Speaking Phrase	Autistic males without ID	4841	65	1.3	12.6	20.4	26.8	28.5	30.4	39.9	64.7
Speaking Phrase	Autistic females with ID	734	126	17.2	13.6	28.0	42.1	46.2	50.7	76.2	156.6
Speaking Phrase	Autistic males with ID	2603	441	16.9	14.8	30.0	44.8	49.0	53.7	80.1	162.1
Acquiring Bladder Control	Siblings without an autism diagnosis or ID	4121	23	0.6	18.5	24.9	29.5	30.6	31.8	37.7	50.8
Acquiring Bladder Control	Autistic females without ID	792	20	2.5	20.7	30.5	38.0	39.9	42.0	52.3	77.1
Acquiring Bladder Control	Autistic males without ID	4067	79	1.9	23.4	32.7	39.5	41.3	43.1	52.1	72.7
Acquiring Bladder Control	Autistic females with ID	691	134	19.4	21.8	38.5	53.1	57.2	61.6	84.9	149.8
Acquiring Bladder Control	Autistic males with ID	2454	382	15.6	23.3	39.1	52.3	55.9	59.8	80.1	134.2
Acquiring Bowel Control	Siblings without an autism diagnosis or ID	4112	35	0.9	19.5	26.3	31.2	32.5	33.8	40.0	54.1
Acquiring Bowel Control	Autistic females without ID	974	39	4.0	21.8	31.9	39.5	41.4	43.5	53.9	78.6
Acquiring Bowel Control	Autistic males without ID	4603	268	5.8	24.5	35.3	43.4	45.6	47.8	58.8	84.8
Acquiring Bowel Control	Autistic females with ID	711	164	23.1	22.8	40.7	56.6	61.0	65.8	91.3	163.3
Acquiring Bowel Control	Autistic males with ID	2529	525	20.8	25.3	43.2	58.5	62.7	67.2	91.0	155.6

© 2022 Kuo SS et al. *JAMA Pediatrics*.

Note. ID: Intellectual disability. Siblings without an autism diagnosis or ID (*n*=4,145); Autistic males with ID (*n*=2,875); Autistic females with ID (*n*=790); Autistic males without ID (*n*=5,000); Autistic females without ID (*n*=1,026). Siblings without an autism diagnosis or ID show comparable developmental milestone attainment compared to the general population[8-10].

© 2022 Kuo SS et al. *JAMA Pediatrics*.

eTable 8. Comparisons of months to milestone attainment grouped by sex and intellectual disability.

Milestone	Comparison	χ2	*df*	Adjusted *p*
Smiling	Siblings without an autism diagnosis or ID / Autistic males with ID	27.185	1	<0.001*
Smiling	Siblings without an autism diagnosis or ID / Autistic males without ID	21.200	1	<0.001*
Smiling	Siblings without an autism diagnosis or ID / Autistic females with ID	23.707	1	<0.001*
Smiling	Siblings without an autism diagnosis or ID / Autistic females without ID	14.625	1	<0.001*
Smiling	Autistic males with ID / Autistic males without ID	7.302	1	<0.001*
Smiling	Autistic males with ID / Autistic females with ID	2.577	1	0.011*
Smiling	Autistic males with ID / Autistic females without ID	4.452	1	<0.001*
Smiling	Autistic males without ID / Autistic females with ID	8.022	1	<0.001*
Smiling	Autistic males without ID / Autistic females without ID	0.075	1	0.951
Smiling	Autistic females with ID / Autistic females without ID	5.785	1	<0.001*
Sitting Upright	Siblings without an autism diagnosis or ID / Autistic males with ID	28.809	1	<0.001*
Sitting Upright	Siblings without an autism diagnosis or ID / Autistic males without ID	15.979	1	<0.001*
Sitting Upright	Siblings without an autism diagnosis or ID / Autistic females with ID	26.849	1	<0.001*
Sitting Upright	Siblings without an autism diagnosis or ID / Autistic females without ID	10.226	1	<0.001*
Sitting Upright	Autistic males with ID / Autistic males without ID	14.433	1	<0.001*
Sitting Upright	Autistic males with ID / Autistic females with ID	4.052	1	<0.001*
Sitting Upright	Autistic males with ID / Autistic females without ID	8.038	1	<0.001*
Sitting Upright	Autistic males without ID / Autistic females with ID	15.805	1	<0.001*
Sitting Upright	Autistic males without ID / Autistic females without ID	0.158	1	0.895
Sitting Upright	Autistic females with ID / Autistic females without ID	9.950	1	<0.001*
Crawling	Siblings without an autism diagnosis or ID / Autistic males with ID	26.771	1	<0.001*
Crawling	Siblings without an autism diagnosis or ID / Autistic males without ID	16.872	1	<0.001*
Crawling	Siblings without an autism diagnosis or ID / Autistic females with ID	26.797	1	<0.001*
Crawling	Siblings without an autism diagnosis or ID / Autistic females without ID	12.600	1	<0.001*
Crawling	Autistic males with ID / Autistic males without ID	10.838	1	<0.001*
Crawling	Autistic males with ID / Autistic females with ID	4.915	1	<0.001*

© 2022 Kuo SS et al. *JAMA Pediatrics*.

Crawling	Autistic males with ID / Autistic females without ID	4.842	1	<0.001*
Crawling	Autistic males without ID / Autistic females with ID	13.691	1	<0.001*
Crawling	Autistic males without ID / Autistic females without ID	1.610	1	0.118
Crawling	Autistic females with ID / Autistic females without ID	7.794	1	<0.001*
Walking	Siblings without an autism diagnosis or ID / Autistic males with ID	29.896	1	<0.001*
Walking	Siblings without an autism diagnosis or ID / Autistic males without ID	19.593	1	<0.001*
Walking	Siblings without an autism diagnosis or ID / Autistic females with ID	29.232	1	<0.001*
Walking	Siblings without an autism diagnosis or ID / Autistic females without ID	15.625	1	<0.001*
Walking	Autistic males with ID / Autistic males without ID	17.318	1	<0.001*
Walking	Autistic males with ID / Autistic females with ID	6.253	1	<0.001*
Walking	Autistic males with ID / Autistic females without ID	7.833	1	<0.001*
Walking	Autistic males without ID / Autistic females with ID	20.275	1	<0.001*
Walking	Autistic males without ID / Autistic females without ID	2.540	1	0.012*
Walking	Autistic females with ID / Autistic females without ID	11.381	1	<0.001*
Spoon-Feeding Self	Siblings without an autism diagnosis or ID / Autistic males with ID	37.498	1	<0.001*
Spoon-Feeding Self	Siblings without an autism diagnosis or ID / Autistic males without ID	22.658	1	<0.001*
Spoon-Feeding Self	Siblings without an autism diagnosis or ID / Autistic females with ID	29.515	1	<0.001*
Spoon-Feeding Self	Siblings without an autism diagnosis or ID / Autistic females without ID	10.434	1	<0.001*
Spoon-Feeding Self	Autistic males with ID / Autistic males without ID	19.556	1	<0.001*
Spoon-Feeding Self	Autistic males with ID / Autistic females with ID	0.709	1	0.501
Spoon-Feeding Self	Autistic males with ID / Autistic females without ID	12.903	1	<0.001*
Spoon-Feeding Self	Autistic males without ID / Autistic females with ID	14.280	1	<0.001*
Spoon-Feeding Self	Autistic males without ID / Autistic females without ID	3.765	1	<0.001*
Spoon-Feeding Self	Autistic females with ID / Autistic females without ID	11.481	1	<0.001*
Speaking Word	Siblings without an autism diagnosis or ID / Autistic males with ID	43.574	1	<0.001*
Speaking Word	Siblings without an autism diagnosis or ID / Autistic males without ID	34.968	1	<0.001*
Speaking Word	Siblings without an autism diagnosis or ID / Autistic females with ID	35.112	1	<0.001*
Speaking Word	Siblings without an autism diagnosis or ID / Autistic females without ID	21.932	1	<0.001*
Speaking Word	Autistic males with ID / Autistic males without ID	24.735	1	<0.001*

© 2022 Kuo SS et al. *JAMA Pediatrics*.

Speaking Word	Autistic males with ID / Autistic females with ID	0.904	1	0.388
Speaking Word	Autistic males with ID / Autistic females without ID	15.516	1	<0.001*
Speaking Word	Autistic males without ID / Autistic females with ID	16.925	1	<0.001*
Speaking Word	Autistic males without ID / Autistic females without ID	3.901	1	<0.001*
Speaking Word	Autistic females with ID / Autistic females without ID	12.898	1	<0.001*
Speaking Phrase	Siblings without an autism diagnosis or ID / Autistic males with ID	54.153	1	<0.001*
Speaking Phrase	Siblings without an autism diagnosis or ID / Autistic males without ID	41.625	1	<0.001*
Speaking Phrase	Siblings without an autism diagnosis or ID / Autistic females with ID	44.048	1	<0.001*
Speaking Phrase	Siblings without an autism diagnosis or ID / Autistic females without ID	27.054	1	<0.001*
Speaking Phrase	Autistic males with ID / Autistic males without ID	37.068	1	<0.001*
Speaking Phrase	Autistic males with ID / Autistic females with ID	1.281	1	0.214
Speaking Phrase	Autistic males with ID / Autistic females without ID	22.044	1	<0.001*
Speaking Phrase	Autistic males without ID / Autistic females with ID	25.829	1	<0.001*
Speaking Phrase	Autistic males without ID / Autistic females without ID	5.162	1	<0.001*
Speaking Phrase	Autistic females with ID / Autistic females without ID	18.503	1	<0.001*
Acquiring Bladder Control	Siblings without an autism diagnosis or ID / Autistic males with ID	49.602	1	<0.001*
Acquiring Bladder Control	Siblings without an autism diagnosis or ID / Autistic males without ID	37.223	1	<0.001*
Acquiring Bladder Control	Siblings without an autism diagnosis or ID / Autistic females with ID	40.478	1	<0.001*
Acquiring Bladder Control	Siblings without an autism diagnosis or ID / Autistic females without ID	24.755	1	<0.001*
Acquiring Bladder Control	Autistic males with ID / Autistic males without ID	29.424	1	<0.001*
Acquiring Bladder Control	Autistic males with ID / Autistic females with ID	1.366	1	0.186
Acquiring Bladder Control	Autistic males with ID / Autistic females without ID	15.324	1	<0.001*
Acquiring Bladder Control	Autistic males without ID / Autistic females with ID	22.478	1	<0.001*
Acquiring Bladder Control	Autistic males without ID / Autistic females without ID	0.058	1	0.954
Acquiring Bladder Control	Autistic females with ID / Autistic females without ID	13.905	1	<0.001*
Acquiring Bowel Control	Siblings without an autism diagnosis or ID / Autistic males with ID	51.388	1	<0.001*
Acquiring Bowel Control	Siblings without an autism diagnosis or ID / Autistic males without ID	40.206	1	<0.001*
Acquiring Bowel Control	Siblings without an autism diagnosis or ID / Autistic females with ID	40.100	1	<0.001*
Acquiring Bowel Control	Siblings without an autism diagnosis or ID / Autistic females without ID	23.908	1	<0.001*

© 2022 Kuo SS et al. *JAMA Pediatrics*.

Acquiring Bowel Control	Autistic males with ID / Autistic males without ID	28.414	1	<0.001*
Acquiring Bowel Control	Autistic males with ID / Autistic females with ID	0.498	1	0.640
Acquiring Bowel Control	Autistic males with ID / Autistic females without ID	19.340	1	<0.001*
Acquiring Bowel Control	Autistic males without ID / Autistic females with ID	18.295	1	<0.001*
Acquiring Bowel Control	Autistic males without ID / Autistic females without ID	5.303	1	<0.001*
Acquiring Bowel Control	Autistic females with ID / Autistic females without ID	15.642	1	<0.001*

Note. Asterisk denotes significant FDR-adjusted p-value<0.05. ID: Intellectual disability. Siblings without an autism diagnosis or ID (n=4,145); Autistic males with ID (n=2,875); Autistic females with ID (n=790); Autistic males without ID (n=5,000); Autistic females without ID (n=1,026). Siblings without an autism diagnosis or ID show comparable developmental milestone attainment compared to the general population[8-10].

© 2022 Kuo SS et al. *JAMA Pediatrics*.

eTable 9. Percentiles of months to milestone attainment grouped by genetic etiology.

Milestone	Group	*n*	Censored	% Censored	5%	25%	45%	50%	55%	75%	95%
Smiling	Autism with known rare variant	194	1	0.5	0.8	1.6	2.4	2.6	2.9	4.3	8.5
Smiling	Autism without known rare variant	2932	0	0.0	0.8	1.6	2.3	2.5	2.7	4.0	7.9
Smiling	Siblings without an autism diagnosis or ID	4094	0	0.0	0.8	1.3	1.7	1.9	2.0	2.7	4.5
Sitting Upright	Autism with known rare variant	197	0	0.0	3.9	5.7	7.1	7.5	7.9	9.8	14.5
Sitting Upright	Autism without known rare variant	2915	0	0.0	3.5	4.9	5.9	6.1	6.4	7.7	10.6
Sitting Upright	Siblings without an autism diagnosis or ID	4065	1	0.0	3.4	4.5	5.2	5.4	5.6	6.5	8.6
Crawling	Autism with known rare variant	195	1	0.5	5.0	7.9	10.1	10.7	11.4	14.6	22.9
Crawling	Autism without known rare variant	2901	33	1.1	4.2	6.5	8.3	8.7	9.2	11.7	17.9
Crawling	Siblings without an autism diagnosis or ID	4056	6	0.1	4.8	6.2	7.2	7.5	7.7	9.0	11.6
Walking	Autism with known rare variant	351	0	0.0	9.3	13.0	15.7	16.4	17.2	20.8	29.1
Walking	Autism without known rare variant	4900	1	0.0	8.7	11.1	12.7	13.1	13.6	15.6	19.9
Walking	Siblings without an autism diagnosis or ID	4121	4	0.1	8.6	10.4	11.6	11.9	12.2	13.6	16.5
Spoon-Feeding Self	Autism with known rare variant	192	12	6.3	7.0	14.1	21.0	23.0	25.2	37.5	75.8
Spoon-Feeding Self	Autism without known rare variant	2857	56	2.0	7.0	12.2	16.7	17.9	19.3	26.4	46.1
Spoon-Feeding Self	Siblings without an autism diagnosis or ID	3968	5	0.1	7.2	10.1	12.2	12.7	13.3	16.1	22.4
Speaking Word	Autism with known rare variant	338	17	5.0	6.7	13.8	20.6	22.6	24.8	37.1	75.7
Speaking Word	Autism without known rare variant	4729	82	1.7	6.7	12.4	17.5	18.9	20.4	28.8	52.9
Speaking Word	Siblings without an autism diagnosis or ID	3931	3	0.1	6.6	9.2	11.2	11.7	12.3	14.9	21.0
Speaking Phrase	Autism with known rare variant	325	33	10.2	13.3	25.1	36.0	39.2	42.5	61.0	115.6
Speaking Phrase	Autism without known rare variant	4623	228	4.9	12.4	22.0	30.5	32.8	35.4	48.9	87.0
Speaking Phrase	Siblings without an autism diagnosis or ID	3975	8	0.2	10.2	14.1	17.0	17.7	18.5	22.2	30.7
Acquiring Bladder Control	Autism with known rare variant	297	43	14.5	22.6	37.3	49.4	52.7	56.3	74.6	123.0
Acquiring Bladder Control	Autism without known rare variant	4436	244	5.5	24.0	34.7	42.8	44.9	47.1	58.1	84.1
Acquiring Bladder Control	Siblings without an autism diagnosis or ID	4121	23	0.6	18.5	24.9	29.5	30.6	31.8	37.7	50.8
Acquiring Bowel Control	Autism with known rare variant	298	53	17.8	23.7	39.8	53.3	57.0	61.0	81.7	137.0
Acquiring Bowel Control	Autism without known rare variant	4405	347	7.9	25.2	37.2	46.4	48.8	51.3	64.0	94.4
Acquiring Bowel Control	Siblings without an autism diagnosis or ID	4112	35	0.9	19.5	26.3	31.2	32.5	33.8	40.0	54.1

Note. ID: Intellectual disability. Autism without known rare variant associated with neurodevelopmental disorders (*n*=4,943); Autism with known rare variant associated with neurodevelopmental disorders (*n*=352). Participants from AGRE, SPARK, and Simons Simplex Collection cohorts.

© 2022 Kuo SS et al. *JAMA Pediatrics*.

eTable 10. Comparisons of months to milestone attainment grouped by genetic etiology.

Milestone	Comparison	$\chi2$	*df*	Adjusted *p*
Smiling	Siblings without an autism diagnosis or ID / Autism with known rare variant	9.942	1	<0.001*
Smiling	Siblings without an autism diagnosis or ID / Autism without known rare variant	20.271	1	<0.001*
Smiling	Autism with known rare variant / Autism without known rare variant	0.971	1	0.331
Sitting Upright	Siblings without an autism diagnosis or ID / Autism with known rare variant	17.803	1	<0.001*
Sitting Upright	Siblings without an autism diagnosis or ID / Autism without known rare variant	18.102	1	<0.001*
Sitting Upright	Autism with known rare variant / Autism without known rare variant	8.335	1	<0.001*
Crawling	Siblings without an autism diagnosis or ID / Autism with known rare variant	19.377	1	<0.001*
Crawling	Siblings without an autism diagnosis or ID / Autism without known rare variant	20.054	1	<0.001*
Crawling	Autism with known rare variant / Autism without known rare variant	8.123	1	<0.001*
Walking	Siblings without an autism diagnosis or ID / Autism with known rare variant	27.509	1	<0.001*
Walking	Siblings without an autism diagnosis or ID / Autism without known rare variant	21.345	1	<0.001*
Walking	Autism with known rare variant / Autism without known rare variant	15.839	1	<0.001*
Spoon-Feeding Self	Siblings without an autism diagnosis or ID / Autism with known rare variant	21.503	1	<0.001*
Spoon-Feeding Self	Siblings without an autism diagnosis or ID / Autism without known rare variant	29.7	1	<0.001*
Spoon-Feeding Self	Autism with known rare variant / Autism without known rare variant	5.705	1	<0.001*
Speaking Word	Siblings without an autism diagnosis or ID / Autism with known rare variant	27.861	1	<0.001*
Speaking Word	Siblings without an autism diagnosis or ID / Autism without known rare variant	37.415	1	<0.001*
Speaking Word	Autism with known rare variant / Autism without known rare variant	5.216	1	<0.001*
Speaking Phrase	Siblings without an autism diagnosis or ID / Autism with known rare variant	33.934	1	<0.001*
Speaking Phrase	Siblings without an autism diagnosis or ID / Autism without known rare variant	46.082	1	<0.001*
Speaking Phrase	Autism with known rare variant / Autism without known rare variant	4.778	1	<0.001*
Acquiring Bladder Control	Siblings without an autism diagnosis or ID / Autism with known rare variant	29.363	1	<0.001*
Acquiring Bladder Control	Siblings without an autism diagnosis or ID / Autism without known rare variant	43.425	1	<0.001*
Acquiring Bladder Control	Autism with known rare variant / Autism without known rare variant	6.693	1	<0.001*
Acquiring Bowel Control	Siblings without an autism diagnosis or ID / Autism with known rare variant	29.455	1	<0.001*
Acquiring Bowel Control	Siblings without an autism diagnosis or ID / Autism without known rare variant	44.301	1	<0.001*
Acquiring Bowel Control	Autism with known rare variant / Autism without known rare variant	6.118	1	<0.001*

© 2022 Kuo SS et al. *JAMA Pediatrics*.

Note. Asterisk denotes significant FDR-adjusted *p*-value<0.05. ID: Intellectual disability. Autism without known rare variant associated with neurodevelopmental disorders (*n*=4,943); Autism with known rare variant associated with neurodevelopmental disorders (*n*=352). Participants from AGRE, SPARK, and Simons Simplex Collection cohorts.

© 2022 Kuo SS et al. *JAMA Pediatrics*.

eTable 11. Percentiles of months to milestone attainment grouped by age at autism diagnosis.

Milestone	Group	*n*	Censored	% Censored	5%	25%	45%	50%	55%	75%	95%
Smiling	Autism diagnosed by age 5 years	6741	3	0.0	0.8	1.6	2.4	2.7	2.9	4.4	9.0
Smiling	Autism diagnosed at ages 5-9 years	2715	2	0.1	0.8	1.6	2.3	2.6	2.8	4.1	8.1
Smiling	Autism diagnosed after age 10 years	692	0	0.0	0.8	1.6	2.3	2.5	2.8	4.0	8.0
Smiling	Siblings without an autism diagnosis or ID	4094	0	0.0	0.8	1.3	1.7	1.9	2.0	2.7	4.5
Sitting Upright	Autism diagnosed by age 5 years	6678	2	0.0	3.5	5.0	6.1	6.4	6.7	8.2	11.6
Sitting Upright	Autism diagnosed at ages 5-9 years	2710	0	0.0	3.4	4.9	5.9	6.2	6.5	7.9	11.2
Sitting Upright	Autism diagnosed after age 10 years	687	0	0.0	3.5	4.9	6.0	6.2	6.5	7.9	11.1
Sitting Upright	Siblings without an autism diagnosis or ID	4065	1	0.0	3.4	4.5	5.2	5.4	5.6	6.5	8.6
Crawling	Autism diagnosed by age 5 years	6637	72	1.1	4.3	6.6	8.4	8.9	9.4	12.0	18.5
Crawling	Autism diagnosed at ages 5-9 years	2686	32	1.2	4.2	6.5	8.3	8.8	9.3	11.9	18.5
Crawling	Autism diagnosed after age 10 years	689	7	1.0	4.1	6.4	8.3	8.8	9.3	12.0	18.8
Crawling	Siblings without an autism diagnosis or ID	4056	6	0.1	4.8	6.2	7.2	7.5	7.7	9.0	11.6
Walking	Autism diagnosed by age 5 years	6788	8	0.1	8.3	11.1	13.1	13.6	14.1	16.7	22.3
Walking	Autism diagnosed at ages 5-9 years	2760	3	0.1	8.2	11.0	13.0	13.5	14.0	16.6	22.3
Walking	Autism diagnosed after age 10 years	700	0	0.0	8.4	11.0	12.7	13.2	13.7	15.9	20.8
Walking	Siblings without an autism diagnosis or ID	4121	4	0.1	8.6	10.4	11.6	11.9	12.2	13.6	16.5
Spoon-Feeding Self	Autism diagnosed by age 5 years	6616	242	3.7	6.8	12.7	18.1	19.6	21.2	30.1	56.0
Spoon-Feeding Self	Autism diagnosed at ages 5-9 years	2633	25	0.9	6.8	11.3	14.9	15.9	17.0	22.6	37.2
Spoon-Feeding Self	Autism diagnosed after age 10 years	670	0	0.0	7.1	10.8	13.7	14.4	15.2	19.3	29.4
Spoon-Feeding Self	Siblings without an autism diagnosis or ID	3968	5	0.1	7.2	10.1	12.2	12.7	13.3	16.1	22.4
Speaking Word	Autism diagnosed by age 5 years	6615	322	4.9	6.3	12.6	18.8	20.5	22.5	33.4	67.2
Speaking Word	Autism diagnosed at ages 5-9 years	2639	20	0.8	6.1	10.4	14.0	15.0	16.1	21.7	36.9
Speaking Word	Autism diagnosed after age 10 years	670	3	0.4	5.9	9.8	13.1	13.9	14.9	19.9	33.2
Speaking Word	Siblings without an autism diagnosis or ID	3931	3	0.1	6.6	9.2	11.2	11.7	12.3	14.9	21.0
Speaking Phrase	Autism diagnosed by age 5 years	6578	826	12.6	12.4	24.3	35.6	38.8	42.3	61.8	120.9
Speaking Phrase	Autism diagnosed at ages 5-9 years	2667	48	1.8	9.9	16.9	22.8	24.4	26.1	35.3	59.9
Speaking Phrase	Autism diagnosed after age 10 years	670	6	0.9	9.3	15.5	20.6	22.0	23.5	31.4	52.1
Speaking Phrase	Siblings without an autism diagnosis or ID	3975	8	0.2	10.2	14.1	17.0	17.7	18.5	22.2	30.7
Acquiring Bladder Control	Autism diagnosed by age 5 years	6714	885	13.2	23.2	36.5	47.2	50.1	53.2	68.8	108.4
Acquiring Bladder Control	Autism diagnosed at ages 5-9 years	2767	93	3.4	20.1	30.1	37.8	39.9	42.0	52.8	79.0
Acquiring Bladder Control	Autism diagnosed after age 10 years	700	6	0.9	17.8	27.2	34.6	36.6	38.6	49.1	75.0
Acquiring Bladder Control	Siblings without an autism diagnosis or ID	4121	23	0.6	18.5	24.9	29.5	30.6	31.8	37.7	50.8
Acquiring Bowel Control	Autism diagnosed by age 5 years	6669	1211	18.2	24.4	39.7	52.2	55.6	59.2	77.9	126.5
Acquiring Bowel Control	Autism diagnosed at ages 5-9 years	2749	130	4.7	21.1	32.2	41.0	43.3	45.8	58.2	89.1
Acquiring Bowel Control	Autism diagnosed after age 10 years	701	9	1.3	19.4	29.8	37.9	40.1	42.3	53.9	82.7
Acquiring Bowel Control	Siblings without an autism diagnosis or ID	4112	35	0.9	19.5	26.3	31.2	32.5	33.8	40.0	54.1

Note. ID: Intellectual disability. Autism diagnosed by age 5 years (*n*=6,859); Autism diagnosed at ages 5-9 years (*n*=2,797); Autism diagnosed after age 10 years (*n*=711); Siblings without an autism diagnosis or ID (*n*=4,145). Participants from SPARK cohort.

© 2022 Kuo SS et al. *JAMA Pediatrics*.

eTable 12. Comparisons of months to milestone attainment grouped by age at autism diagnosis.

Milestone	Comparison	χ2	*df*	Adjusted *p*
Smiling	Siblings without an autism diagnosis or ID / Autism diagnosed by age 5 years	26.912	1	<0.001*
Smiling	Siblings without an autism diagnosis or ID / Autism diagnosed at ages 5-9 years	21.220	1	<0.001*
Smiling	Siblings without an autism diagnosis or ID / Autism diagnosed after age 10 years	15.077	1	<0.001*
Smiling	Autism diagnosed by age 5 years / Autism diagnosed at ages 5-9 years	3.677	1	<0.001*
Smiling	Autism diagnosed by age 5 years / Autism diagnosed after age 10 years	2.151	1	0.036*
Smiling	Autism diagnosed at ages 5-9 years / Autism diagnosed after age 10 years	0.066	1	0.948
Sitting Upright	Siblings without an autism diagnosis or ID / Autism diagnosed by age 5 years	24.940	1	<0.001*
Sitting Upright	Siblings without an autism diagnosis or ID / Autism diagnosed at ages 5-9 years	19.364	1	<0.001*
Sitting Upright	Siblings without an autism diagnosis or ID / Autism diagnosed after age 10 years	12.994	1	<0.001*
Sitting Upright	Autism diagnosed by age 5 years / Autism diagnosed at ages 5-9 years	3.012	1	0.003*
Sitting Upright	Autism diagnosed by age 5 years / Autism diagnosed after age 10 years	2.124	1	0.038*
Sitting Upright	Autism diagnosed at ages 5-9 years / Autism diagnosed after age 10 years	0.340	1	0.748
Crawling	Siblings without an autism diagnosis or ID / Autism diagnosed by age 5 years	24.589	1	<0.001*
Crawling	Siblings without an autism diagnosis or ID / Autism diagnosed at ages 5-9 years	20.516	1	<0.001*
Crawling	Siblings without an autism diagnosis or ID / Autism diagnosed after age 10 years	12.940	1	<0.001*
Crawling	Autism diagnosed by age 5 years / Autism diagnosed at ages 5-9 years	1.181	1	0.251
Crawling	Autism diagnosed by age 5 years / Autism diagnosed after age 10 years	1.629	1	0.114
Crawling	Autism diagnosed at ages 5-9 years / Autism diagnosed after age 10 years	0.900	1	0.382
Walking	Siblings without an autism diagnosis or ID / Autism diagnosed by age 5 years	26.360	1	<0.001*
Walking	Siblings without an autism diagnosis or ID / Autism diagnosed at ages 5-9 years	22.496	1	<0.001*
Walking	Siblings without an autism diagnosis or ID / Autism diagnosed after age 10 years	13.028	1	<0.001*
Walking	Autism diagnosed by age 5 years / Autism diagnosed at ages 5-9 years	1.327	1	0.199
Walking	Autism diagnosed by age 5 years / Autism diagnosed after age 10 years	3.001	1	0.003*
Walking	Autism diagnosed at ages 5-9 years / Autism diagnosed after age 10 years	2.174	1	0.035*
Spoon-Feeding Self	Siblings without an autism diagnosis or ID / Autism diagnosed by age 5 years	36.116	1	<0.001*
Spoon-Feeding Self	Siblings without an autism diagnosis or ID / Autism diagnosed at ages 5-9 years	21.581	1	<0.001*

© 2022 Kuo SS et al. *JAMA Pediatrics*.

Spoon-Feeding Self	Siblings without an autism diagnosis or ID / Autism diagnosed after age 10 years	9.379	1	<0.001*
Spoon-Feeding Self	Autism diagnosed by age 5 years / Autism diagnosed at ages 5-9 years	14.518	1	<0.001*
Spoon-Feeding Self	Autism diagnosed by age 5 years / Autism diagnosed after age 10 years	11.598	1	<0.001*
Spoon-Feeding Self	Autism diagnosed at ages 5-9 years / Autism diagnosed after age 10 years	4.757	1	<0.001*
Speaking Word	Siblings without an autism diagnosis or ID / Autism diagnosed by age 5 years	39.314	1	<0.001*
Speaking Word	Siblings without an autism diagnosis or ID / Autism diagnosed at ages 5-9 years	21.404	1	<0.001*
Speaking Word	Siblings without an autism diagnosis or ID / Autism diagnosed after age 10 years	11.733	1	<0.001*
Speaking Word	Autism diagnosed by age 5 years / Autism diagnosed at ages 5-9 years	19.323	1	<0.001*
Speaking Word	Autism diagnosed by age 5 years / Autism diagnosed after age 10 years	12.381	1	<0.001*
Speaking Word	Autism diagnosed at ages 5-9 years / Autism diagnosed after age 10 years	2.907	1	0.004*
Speaking Phrase	Siblings without an autism diagnosis or ID / Autism diagnosed by age 5 years	50.123	1	<0.001*
Speaking Phrase	Siblings without an autism diagnosis or ID / Autism diagnosed at ages 5-9 years	27.648	1	<0.001*
Speaking Phrase	Siblings without an autism diagnosis or ID / Autism diagnosed after age 10 years	14.470	1	<0.001*
Speaking Phrase	Autism diagnosed by age 5 years / Autism diagnosed at ages 5-9 years	27.293	1	<0.001*
Speaking Phrase	Autism diagnosed by age 5 years / Autism diagnosed after age 10 years	16.953	1	<0.001*
Speaking Phrase	Autism diagnosed at ages 5-9 years / Autism diagnosed after age 10 years	3.901	1	<0.001*
Acquiring Bladder Control	Siblings without an autism diagnosis or ID / Autism diagnosed by age 5 years	47.515	1	<0.001*
Acquiring Bladder Control	Siblings without an autism diagnosis or ID / Autism diagnosed at ages 5-9 years	29.449	1	<0.001*
Acquiring Bladder Control	Siblings without an autism diagnosis or ID / Autism diagnosed after age 10 years	16.713	1	<0.001*
Acquiring Bladder Control	Autism diagnosed by age 5 years / Autism diagnosed at ages 5-9 years	21.258	1	<0.001*
Acquiring Bladder Control	Autism diagnosed by age 5 years / Autism diagnosed after age 10 years	14.321	1	<0.001*
Acquiring Bladder Control	Autism diagnosed at ages 5-9 years / Autism diagnosed after age 10 years	3.708	1	<0.001*
Acquiring Bowel Control	Siblings without an autism diagnosis or ID / Autism diagnosed by age 5 years	49.316	1	<0.001*
Acquiring Bowel Control	Siblings without an autism diagnosis or ID / Autism diagnosed at ages 5-9 years	31.041	1	<0.001*
Acquiring Bowel Control	Siblings without an autism diagnosis or ID / Autism diagnosed after age 10 years	18.425	1	<0.001*
Acquiring Bowel Control	Autism diagnosed by age 5 years / Autism diagnosed at ages 5-9 years	21.710	1	<0.001*
Acquiring Bowel Control	Autism diagnosed by age 5 years / Autism diagnosed after age 10 years	14.838	1	<0.001*
Acquiring Bowel Control	Autism diagnosed at ages 5-9 years / Autism diagnosed after age 10 years	3.604	1	<0.001*

© 2022 Kuo SS et al. *JAMA Pediatrics*.

Note. Asterisk denotes significant FDR-adjusted *p*-value<0.05. ID: Intellectual disability. Autism diagnosed by age 5 years (*n*=6,859); Autism diagnosed at ages 5-9 years (*n*=2,797); Autism diagnosed after age 10 years (*n*=711); Siblings without an autism diagnosis or ID (*n*=4,145). Participants from SPARK cohort.

© 2022 Kuo SS et al. *JAMA Pediatrics*.

eReferences

1. Geschwind DH, Sowinski J, Lord C, et al. The Autism Genetic Resource Exchange: A resource for the study of autism and related neuropsychiatric conditions. *Am J Hum Genet*. Aug 2001;69(2):463-6. doi:10.1086/321292
2. Lajonchere CM, Consortium A. Changing the landscape of autism research: The Autism Genetic Resource Exchange. *Neuron*. Oct 21 2010;68(2):187-91. doi:10.1016/j.neuron.2010.10.009
3. Buxbaum JD, Bolshakova N, Brownfeld JM, et al. The Autism Simplex Collection: An international, expertly phenotyped autism sample for genetic and phenotypic analyses. *Mol Autism*. 2014/05/20 2014;5(1):34. doi:10.1186/2040-2392-5-34
4. Fischbach GD, Lord C. The Simons Simplex Collection: A resource for identification of autism genetic risk factors. *Neuron*. Oct 21 2010;68(2):192-5. doi:10.1016/j.neuron.2010.10.006
5. The SPARK Consortium. SPARK: A US cohort of 50,000 families to accelerate autism research. *Neuron*. 2018;97(3):488-493.
6. Lord C, Rutter M, Le Couteur A. Autism Diagnostic Interview-Revised: A revised version of a diagnostic interview for caregivers of individuals with possible pervasive developmental disorders. *J Autism Dev Disord*. Oct 1994;24(5):659-85. doi:10.1007/BF02172145
7. Lord C, Risi S, Lambrecht L, et al. The Autism Diagnostic Observation Schedule-Generic: A standard measure of social and communication deficits associated with the spectrum of autism. *J Autism Dev Disord*. Jun 2000;30(3):205-23.
8. Schum TR, Kolb TM, McAuliffe TL, Simms MD, Underhill RL, Lewis M. Sequential acquisition of toilet-training skills: A descriptive study of gender and age differences in normal children. *Pediatrics*. 2002;109(3):e48-e48. doi:10.1542/peds.109.3.e48
9. Frankenburg WK, Dodds J, Archer P, Shapiro H, Bresnick B. The Denver II: a major revision and restandardization of the Denver Developmental Screening Test. *Pediatrics*. Jan 1992;89(1):91-7.
10. WHO Multicentre Growth Reference Study Group. WHO Motor Development Study: windows of achievement for six gross motor development milestones. *Acta Paediatr Suppl*. Apr 2006;450:86-95. doi:10.1111/j.1651-2227.2006.tb02379.x

© 2022 Kuo SS et al. *JAMA Pediatrics*.
